# Autophagy, a Conserved Mechanism for Protein Degradation, Responds to Heat, and Other Abiotic Stresses in *Capsicum annuum* L.

**DOI:** 10.3389/fpls.2016.00131

**Published:** 2016-02-09

**Authors:** Yufei Zhai, Meng Guo, Hu Wang, Jinping Lu, Jinhong Liu, Chong Zhang, Zhenhui Gong, Minghui Lu

**Affiliations:** Department of Vegetable Science, College of Horticulture, Northwest A&F UniversityYangling, China

**Keywords:** *Capsicum annuum* L., autophagy-related genes, genome-wide identification, heat stress, expression profiles

## Abstract

Abiotic stresses negatively affect plants growth and development by inducing protein denaturation, and autophagy degrades the damaged proteins to alleviate their toxicity, however, little is known about the involvement of autophagy in pepper (*Capsicum annuum* L.) tolerances to abiotic stresses. In this study, we identified autophagy-related gene (*ATG*) members in the whole genome of pepper by HMM method and analyzed their expression profiles in response to heat and other abiotic stresses by quantitative real-time PCR. The results showed that the CaATG contained 15 core ATG members including 29 ATG proteins with their respective conserved functional domains, involving the whole process of autophagy. Under normal environmental condition, the expression of *CaATG* genes showed tissue- and developmental stage-specific patterns, while under abiotic stresses of salt, drought, heat, cold and carbohydrate starvation, the accumulation of autophagosome punctate increased and the expression level of *CaATG* genes changed with stress type-dependent pattern, which indicates the linkage of autophagy in pepper response to abiotic stresses. After treated with heat stress, both the number of up-regulated *CaATG* genes and the increment of autophagosome punctate were higher in pepper thermotolerant line R9 than those in thermosensitive line B6, implying an association of autophagy with heat tolerance. In addition, CaATG6 was predicted to interact with CaHSP90 family members. Our study suggests that autophagy is connected to pepper tolerances to heat and other abiotic stresses.

## Introduction

In general, plants grow in an open ecosystem with the sessile lifestyle, which makes them routinely suffer from adverse environmental conditions, mainly including high and low temperature, drought, salt, and starvation stress (Wang et al., 2003; Ahuja et al., 2010; Wang and Liu, 2010). With the aggravating of global warming in recent years, extreme high temperature climate occurs more frequently (IPCC, 2013), and it is estimated that 1°C rise in temperature could lowers the yield up to 10% (Lobell et al., 2011), thus heat stress becomes a major threat for crop production.

High temperature negatively affects plants growth and development by inducing protein misfolding, denaturation, oxidation, and aggregation (Hemantaranjan et al., 2014). Organisms develop two types of mechanisms in evolution to alleviate the injury from heat stress, i.e., effectively preventing the proteins to be damaged and efficiently removing the damaged proteins (Dokladny et al., 2015). In plants, the major executors to protect proteins under heat stress are heat shock proteins (HSPs) as molecular chaperones (Liu et al., 2012), which was verified by the studies of our research group and others in pepper (*Capsicum annuum* L.) (Zhu et al., 2011; Guo et al., 2014a,b), tomato (*Solanum lycopersicum* L.) (Bita et al., 2011), wheat (*Triticum aestivum* L.) (Xue et al., 2014), and cucumber (*Cucumis sativus* L.) (Li et al., 2014). However, the relationship between the ability to remove damaged proteins and thermotolerance of plants is still unclear and remained to be studied.

The removal of damaged proteins can be conducted by autophagy, one of the ubiquitous and highly conservative protein degradation systems in eukaryotic cells (Yoshimoto et al., 2010). During the autophagy process, the damaged proteins and organelles were wrapped into double-membrane vesicle called autophagosome and then transported into vacuoles for breakdown (Johansen and Lamark, 2011; Liu and Bassham, 2012). Recent studies divulged that autophagy participated in plant responses to environmental stresses like salt, drought, cold, oxdative, hypoxia, and osmotic stress (Slavikova et al., 2008; Liu et al., 2009; Shin et al., 2009; Kuzuoglu-Ozturk et al., 2012; Pei et al., 2014; Chen et al., 2015).

As for heat stress, although Zhou et al. (2013, 2014) found functional loss or silent expression of autophagy related genes (ATG) *ATG5, ATG7*, and their regulator *NBR1* (neighbor of BRCA1) decreased the thermotolerance of *Arabidopsis* and tomato by accumulating more insoluble protein, but systematic analysis of *ATGs* in plant response to heat stress is limited. In our current study, 15 putative ATG members including 29 ATG proteins were identified in pepper, a temperate but thermosensitve vegetable crop, and their expression profiles in response to heat stress and other abiotic stresses were comparatively analyzed, aiming to provide a systematic base for further studies on the function and regulation of autophagy in the formation of plant thermotolerance.

## Materials and methods

### Identification of CaATG homologs

The identification of putative CaATG proteins was performed by the HMMER 3.0 software, in which the HMM profiles of pfam (http://pfam.xfam.org/) for each ATG member were generated from the published amino acid sequences of ATG members of *Arabidopsis* (Hanaoka et al., 2002; Kwon and Park, 2008) and rice (Xia et al., 2011), and pepper genomic protein sequences were downloaded from database PGP (V1.55) (http://peppergenome.snu.ac.kr) (Kim et al., 2014) and PGD (V2.0) (http://peppersequence.genomics.cn/page/species/index.jsp) (Qin et al., 2014). All output protein sequences were collected and then confirmed by the SMART (http://smart.embl-heidelberg.de/) and blastp program in NCBI (http://www.ncbi.nlm.nih.gov/). The candidate CaATG protein sequences from the three pepper cultivars (CM334, Zunla-1 and Chiltepin) were aligned by ClustalW2 online software (http://www.ebi.ac.uk/Tools/msa/clustalw2/) to determine the protein sequences, which were confirmed again by operating blastp. The theoretical iso-electric point (pI) and molecular weight (MW) of each CaATG protein were calculated by Compute pI/Mw tool (http://web.expasy.org/compute_pi/), and the proteins subcellular location were predicted in online software WoLF PSORT (http://wolfpsort.seq.cbrc.jp/).

### Phylogenetic analysis

The software MEGA 6 (Tamura et al., 2013) was recruited to construct the phylogenetic trees of the full-length of ATG protein sequences from pepper, rice, *Arabidopsis*, algae and yeast, in which the neighbor-joining method, *p*-distance substitution model and 1000 bootstrap replicates were selected.

### Sequence structure, chromosomal location, and conserved motifs analysis

The exon/intron structures of *CaATG* genes were determined based on alignments of coding sequences with their respective genomic full-length sequences (http://peppergenome.snu.ac.kr), and the structural diagrams were generated by the online program Gene Structure Display Server (GSDS, http://gsds.cbi.pku.edu.ch). MapInspect software (Ralph van Berloo, Laboratory of Plant Breeding, Wageningen University) was used to draw the chromosomal locations of *CaATG* genes.

Conserved motifs of the CaATG protein sequences were identified by MEME program (http://meme.nbcr.net/meme/), in which normal motif discovery mode and 20 for maximum number of motifs were selected, and the parameter of site distribution was set as zero or one occurrence per sequence.

### RNA-Seq analysis of *CaATG* genes tissue-specific expression

Based on the databases of genomic RNA-seq (Kim et al., 2014), the tissue and stage-specific expression profiles of 26 *CaATG* genes (the data of *CaATG5, ATG12*, and *VPS34* are absent) were analyzed in 17 tissues of pepper cultivar “CM334” and seven tissues of “ECW30R,” including root, stem, leaf, pericarp and placenta at different days post-anthesis (PC-6DPA, -16DPA and -25DPA, PL-6DPA, PL-13DPA, -16DPA, PL-20DPA, and-25DPA), pericarp and placenta at mature green (PC-MG, PL-MG) and at breaker (PC-B, PL-B), pericarp and placenta at 5 and 10 days post-breaker (PC-B5 and PC-B10, PL-B5 and PL-B10).

### Heat and other abiotic stresses treatments

The pepper thermotolerant line R9 (sweet pepper, introduced from the World-Asia Vegetable Research and Development Center, PP0042-51) and thermosensitive line B6 (hot pepper, selected by the pepper research group, College of Horticulture, Northwest A&F University, Yangling, China) were used in this study. The pepper seedlings were grown under normal conditions (26°C/20°C day/night, photoperiod of 16 h light/8 h dark cycle, 200 μmol·m^−2^·s^−1^ illumination intensity and 70% relative humidity) in a controlled climate chamber.

Pepper seedlings at 5–6 true leaves stage were used for abiotic stresses treatments. The seedlings of control treatment were grown under normal conditions, while for heat stress (HS) treatments, seedlings of R9 and B6 were transferred into climate chamber with temperature of 40°C, and the leaves were collected at 0, 1, 3, 6, and 12 h after HS. The seedlings of R9 were kept at 4°C for 3 h for cold treatment, kept in the dark for 2 days for carbohydrate starvation (Pei et al., 2014), incubated in Hoagland's solution containing 200 mM NaCl for 3 h for salt treatment (Xiao et al., 2014), and dried for 3 h between folds of tissue pepper for drought treatment (Xia et al., 2011). The roots and stems of pepper seedlings were collected for NaCl treatment, and the roots, stems and leaves for drought treatment, while the leaves for other treatments. After collection, the samples were immediately frozen in liquid nitrogen and stored at −80°C for RNA extraction. Each treatment was conducted with three biological replicates and each replicate contained five pepper seedlings.

### RNA extraction and qRT-PCR analysis

Total RNA was extracted from frozen samples using Plant RNA Kit (OMEGA) according to the manufacturer's instructions, and then cDNA was generated using the Primescript™first strand cDNA Synthesis Kit (Takara). Quantitative real-time PCR (qRT-PCR) for relative quantification of *CaATGs* expression was performed according to the method of Guo et al. (2014b), and the ubiquitin-conjugating protein gene *UBI-3* (AY486137) was used as the reference gene (Wan et al., 2011). The *CaATGs* gene-specific primer pairs for qRT-PCR were listed in Table S1. The relative expression levels of pepper *CaATG* genes were calculated following the 2^−Δ*ΔCT*^ method (Livak and Schmittgen, 2001), in which the relative expression levels of the control seedlings were set to one and two-fold change was looked as the standard to judge the up- or down-regulation. The significance test for difference in expression levels of each *CaATG* gene between control and stress treatment was performed by student's *t*-test method at α = 0.01 level.

### Generation of the heatmap for gene expression level

The heatmaps for *CaATG* genes expression level were created by HemI (The Cuckoo Workgroup, Wuhan, China). For the tissue-specific expression, the RNA-seq data extracted from the published databases of Kim et al. (2014) were normalized using logarithm with the base of 2, and those genes having no transcription were indicated as white color. For the expression under abiotic or heat stress, the values of 2^−Δ*ΔCT*^ were used directly for the up-regulated genes, while the values of −(2^−Δ*ΔCT*^)^−1^ were employed for the down-regulated genes to make the heatmap be observed more intuitively.

### Autophagosome staining and microscopic observation

The combination of E-64d for autophagosomes accumulation and LysoTracker for staining have been widely used to detecting autophagic activity in a variety of organisms including plants (Moriyasu et al., 2003; Kuzuoglu-Ozturk et al., 2012; Phadwal et al., 2012; Chikte et al., 2014). After infiltrated with 100 μM E-64d (Sigma), pepper leaves and root segments were stained with 2 μM LysoTracker Red DND-99 (Invitrogen) according to the method of Liu et al. (2005). The stained autophagosome punctates were observed under fluorescent microscope (BX51; Olympus) using a 50-W mercury lamp, and punctate areas were scanned by the tool of particles analysis in ImageJ 1.46 (National Institutes of Health, Bethesda, Maryland). The Statistical Analysis System software (version 8.2, SAS Institute, Cary, NC, USA) for one-way analysis of variance (ANOVA) and the shortest significant ranges (*SSR*) method were used to compare the differences in punctate areas among the leaves of B6 and R9 with and without heat stress at the level α = 0.05.

### Prediction of protein-protein interaction network

The interolog from *Arabidopsis* was used for predicting protein-protein interaction network among CaATG members and HSPs by searching in STRING database (Search Tool for the Retrieval of Interacting Genes/Proteins) (http://string-db.org). In order to improve the reliability of prediction results, the parameters with a high confidence (0.700) was set to filter the edge information of all the ATG members as well as HSPs interacting with ATGs. The output was finally imported into Cytoscape_v3.2.1 (National Institute of General Medical Sciences, MD, USA) to generate the protein-protein interaction network map.

## Results

### Genome-wide identification of *ATG* genes in pepper

Since no published data is available about autophagy related genes in pepper so far, the sequences of ATG members from *Arabidopsis* (Hanaoka et al., 2002; Kwon and Park, 2008) and rice (Xia et al., 2011) were used to search the pepper genome database (http://peppergenome.snu.ac.kr). The searching results showed that 15 core ATG members containing 29 proteins with ATG domains were identified (Table 1; Table S2), involving all processes of autophagy.

**Table 1:** **The autophagy-related genes (ATG) homologs in pepper**.

**ATG members**	**ATG proteins**	**Gene ID**	**Chr**.	**Length of protein**	**MW (kDa)**	**pI**	**Predicted localization**	**Predicted function**
CaATG1	CaATG1a	Capang00g000858	08g	335	37.77	6.49	Plasma membrane	Serine/threonine-protein kinase
	CaATG1b	Capang09g001292	09g	716	79.54	6.85	Cytoplasm	Detected protein of unknown function
	CaATG1c	Ca10g14330	10g	719	79.61	5.93	Endoplasmic reticulum (membrane)	Detected protein of unknown function
CaATG2	CaATG2	Capana08g002378	08g	2001	219.80	5.06	Mitochondrial matrix space	Autophagy-related protein
CaATG3	CaATG3	Capana06g002420	06g	297	33.22	4.69	Cytoplasm	Autophagy-related protein 3
CaATG4	CaATG4	Ca01g11770	01g	499	54.39	5.27	Microbody (peroxisome)	Cysteine protease ATG4
CaATG5	CaATG5	Capang02g000018	02g	401	44.62	5.16	Cytoplasm	
CaATG6	CaATG6	Ca05g11470	05g	530	60.03	6.30	Cytoplasm	Beclin 1 protein
CaATG7	CaATG7	Ca11g16250	11g	714	77.97	5.61	Plasma membrane	Autophagy protein, putative
CaATG8	CaATG8a	Ca07g18990	07g	119	13.74	8.86	Cytoplasm	Autophagy 8a
	CaATG8b	Ca02g18300	02g	123	14.10	8.30	Cytoplasm	Autophagy 8b
	CaATG8c	Capana01g004401	01g	122	14.07	8.30	Cytoplasm	Autophagy 8c
	CaATG8d	Ca01g13140	01g	122	13.96	8.28	Cytoplasm	Autophagy 8f
	CaATG8e	Ca04g03430	04g	121	13.95	5.47	Mitochondrial matrix space	Gaba(A) receptor-associated protein, putative
CaATG9	CaATG9	Ca05g06420	05g	865	99.87	6.68	Chloroplast thylakoid membrane	At2g31260/F16D14.10
CaATG10	CaATG10a	Capang00g001180	08g	238	27.10	5.76	Microbody (peroxisome)	Transporter, putative
	CaATG10b	Ca11g16340	11g	230	26.17	4.99	Microbody (peroxisome)	Transporter, putative
CaATG12	CaATG12	Capana12g000969	12g	217	24.17	6.72	Microbody (peroxisome)	
CaATG13	CaATG13a	Ca03g20810	03g	608	67.45	8.58	Microbody (peroxisome)	At3g49590 protein
	CaATG13b	Capana06g000762	06g	607	67.25	7.80	Cytoplasm	Autophagy-related protein
CaATG18	CaATG18a	Ca01g09640	01g	433	47.84	8.45	Cytoplasm	WD-repeat protein, putative
	CaATG18b	Ca01g30560	01g	423	47.06	8.38	Cytoplasm	At3g62770/F26K9_200
	CaATG18c	Ca06g07940	06g	417	45.71	7.77	Chloroplast stroma	WD-repeat protein, putative
	CaATG18d	Capang01g005155	01g	879	94.91	6.78	Cytoplasm	Breast carcinoma amplified sequence, putative
	CaATG18e	Capana08g001383	08g	1038	112.72	5.65	Nucleus	Breast carcinoma amplified sequence, putative
	CaATG18f	Capana00g003665	00g	362	39.28	7.75	Endoplasmic reticulum (membrane)	WD-repeat protein, putative
	CaATG18g	Ca00g77430	00g	450	49.08	6.90	Cytoplasm	At3g62770/F26K9_200
CaVPS15	CaVPS15	Ca05g02690	05g	1552	173.29	6.89	Endoplasmic reticulum (membrane)	Phosphoinositide-3-kinase, regulatory subunit 4
CaVPS34	CaVPS34	Capana05g000146	05g	812	93.21	6.56	Cytoplasm	Phosphoinositide 3-kinase

*In the IDs of CaATG, prefixion of “Capang” were from pepper cultivar “Chiltepin,” “Ca” from “CM334” and “Capana” from “Zunla-1” genome. Chr., chromosomal; MW, molecular weight; pI, isoelectric point*.

Among the 15 ATG members in pepper, there were seven proteins in ATG18 (CaATG18a-18g), five in ATG8 (CaATG8a-8e), three in ATG1 (CaATG1a-1c), two in ATG10 (CaATG10a/10b), and ATG13 (CaATG13a/13b) respectively, and one in each of the other ATG members (ATG2, ATG3, ATG4, ATG5, ATG6, ATG7, ATG9, ATG12, VPS15, and VPS34) (Table 1). The CaATG proteins varied from 119 to 2001 amino acid (aa) in length, from 13.744 to 219.801 kDa in molecular weight and from 4.69 to 8.86 in predicted isoelectric points (Table 1).

The 29 *CaATG* genes were unevenly distributed on the 12 chromosomes of pepper, in which 6 *CaATG* genes were located on Chr01, and 5 genes on Chr08, 4 on Chr05, 3 on Chr06, 2 on Chr02, 07, and 11 respectively, and only 1 on each of the remaining 5 chromosomes respectively (Figure S1).

### Phylogenetic relationship, conserved domains, and sequence structure of *CaATG* genes

**Figure 1:**
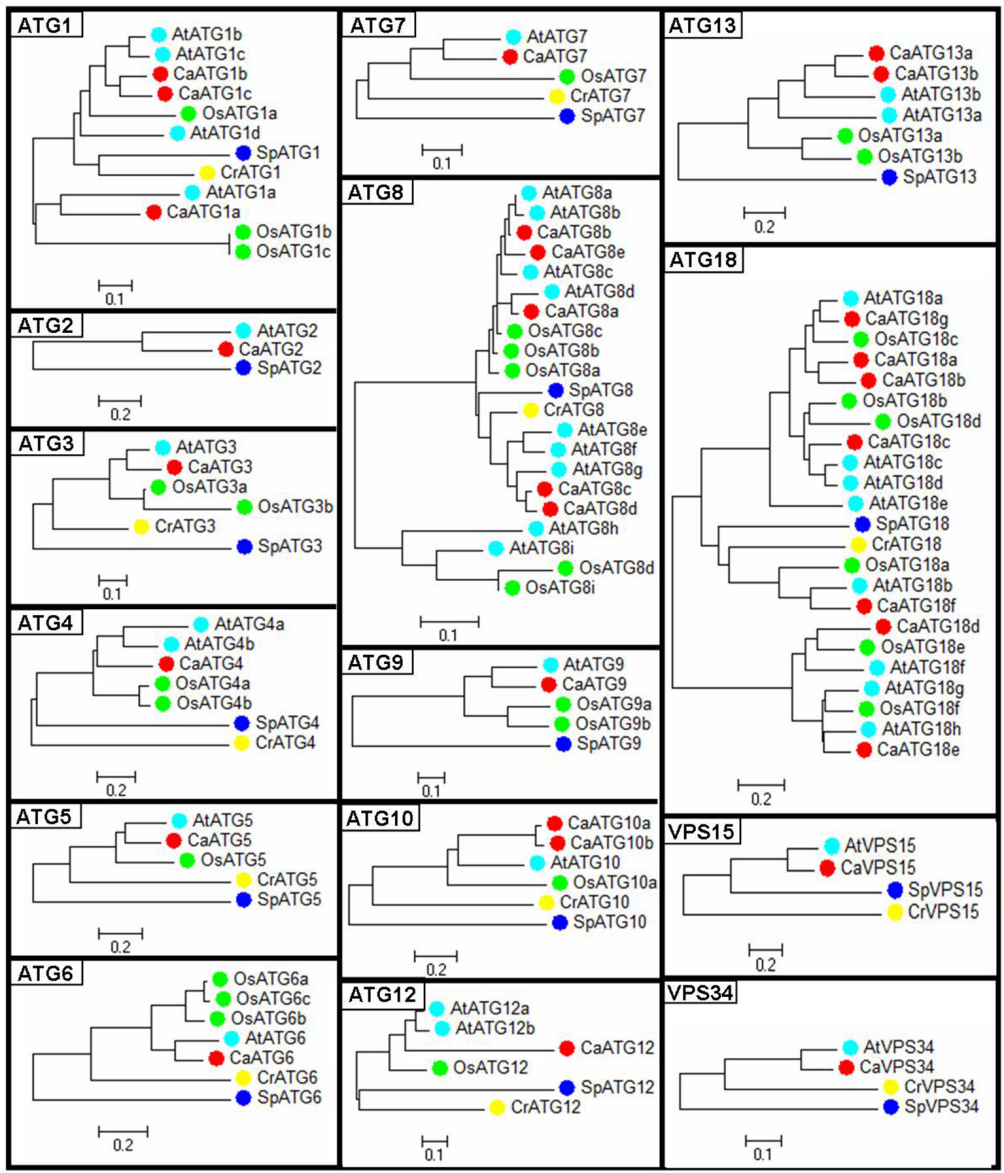
**Phylogenetic tree of ATG proteins from pepper**. Ca, *Capsicum annuum* L.; At, *Arabidopsis thaliana*; Os, *Oryza sativa*; Cr, *Chlamydomonas reinhardtii;* Sp, *Saccharomyces cerevisiae*.

Phylogenetic trees of the 15 ATG members were constructed respectively based on the ATG protein sequences from five species including pepper, *Arabidopsis*, rice, algae and yeast (Figure 1). Compared to those from algae and yeast, the ATG proteins from three higher plant species were basically clustered together. Twenty-six out of twenty-nine pepper ATG proteins (89.6%) except CaATG18a/18b/18d showed the nearest phylogenetic relationship with those of *Arabidopsis*, agreeing with their botanical classifications.

**Figure 2:**
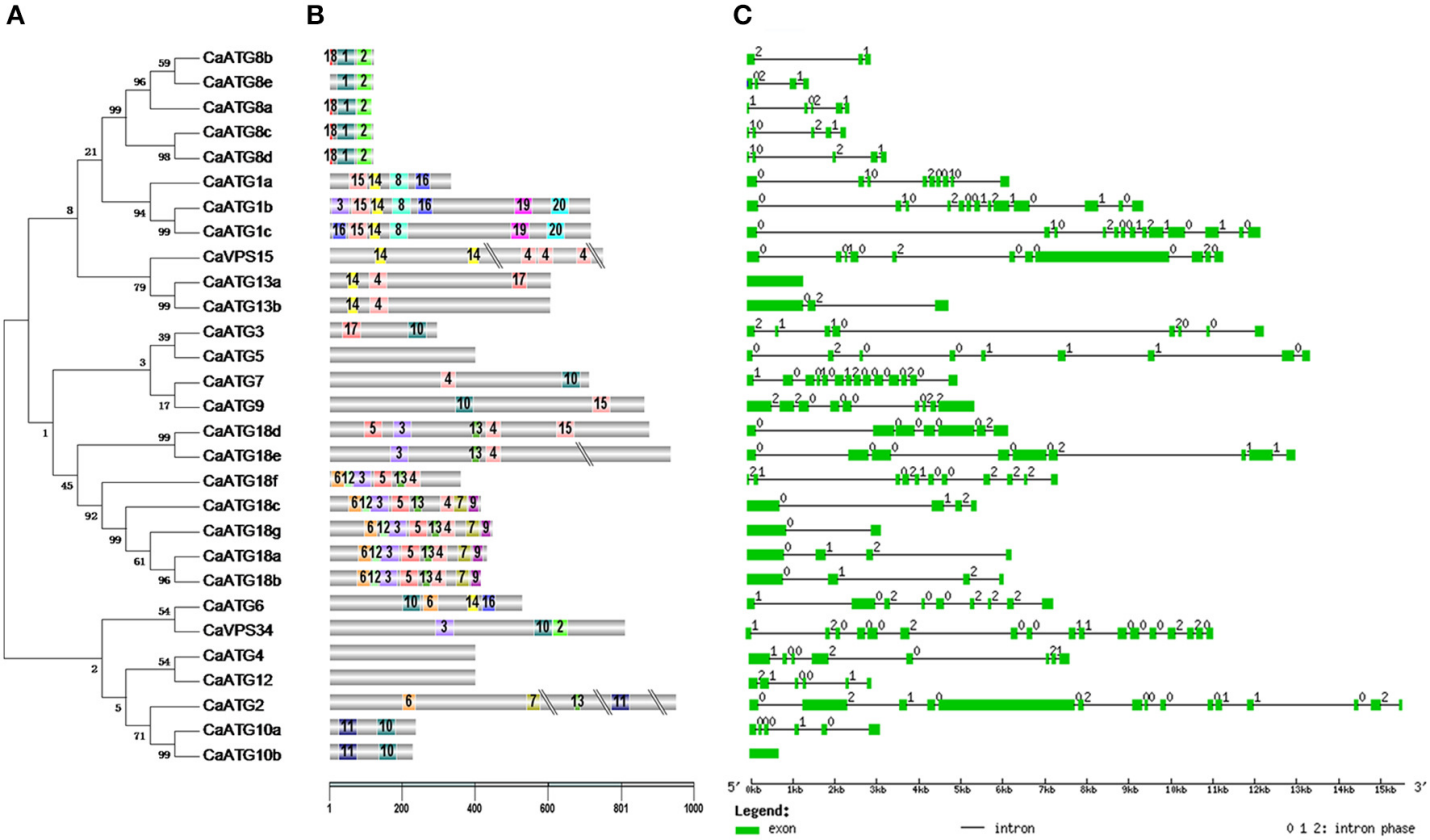
**Conserved domains and sequence structure of *CaATG* genes. (A)** Phylogenetic analysis of CaATG proteins. Unrooted phylogenetic tree built on the basis of full-length of ATG protein sequences; **(B)** Motifs of CaATG proteins identified by MEME tools. Different motifs are indicated by different borders and colors; **(C)** Exon/intron organizations of CaATG genes. Green boxes indicate the exon regions and lines indicate introns. The length of the boxes and lines are scaled based on the length of genes. Numbers 0, 1, and 2 represent introns in phases 0, 1, and 2 respectively.

The classification of 29 CaATG proteins was coincident with the motifs distribution identified by MEME web server (http://meme-suite.org/tools/meme) (Figures 2A,B, Table S3). For instance, the conserved domains of motif1-motif2 and motif13-motif4 were observed in all of the five CaATG8 proteins and all of the eight CaATG18 proteins, respectively. Similar structure of motif15-motif14-motif8 at N-terminal was also presented in CaATG1a, 1b, and 1c. However, diversity in motifs and their distribution were found. For example, CaATG8e did not contain motif18 like the other 4 ATG8 proteins, and CaATG1a lacked the C-terminal structure of motif19-motif20 as shown in ATG1b and c (Figures 2A,B).

The structures in exon/intron distribution *CaATG* genes were further analyzed based on the alignments of their coding region sequences with respective genomic full-length sequences. Except *CaATG10b* and *CaATG13a*, the other 27 *CaATG* genes contained multiple introns with irregular intron phases for 0, 1, and 2 (Figure 2C).

By subcellular localization prediction software WoLF PSORT, it was predicted that 14 of the 29 CaATG proteins were located in the cytoplasm, five in microbody (peroxisome), three in endoplasmic reticulum, two in plasma membrane (CaATG1a and CaATG7) and mitochondrial matrix space (CaATG2 and CaATG8e), and one in chloroplast thylakoid membrane (CaATG9), chloroplast stroma (CaATG18c), and nucleus (CaATG18e), respectively (Table 1).

### RNA-Seq analysis of caatg genes tissue- and developmental stage-specific expression

**Figure 3:**
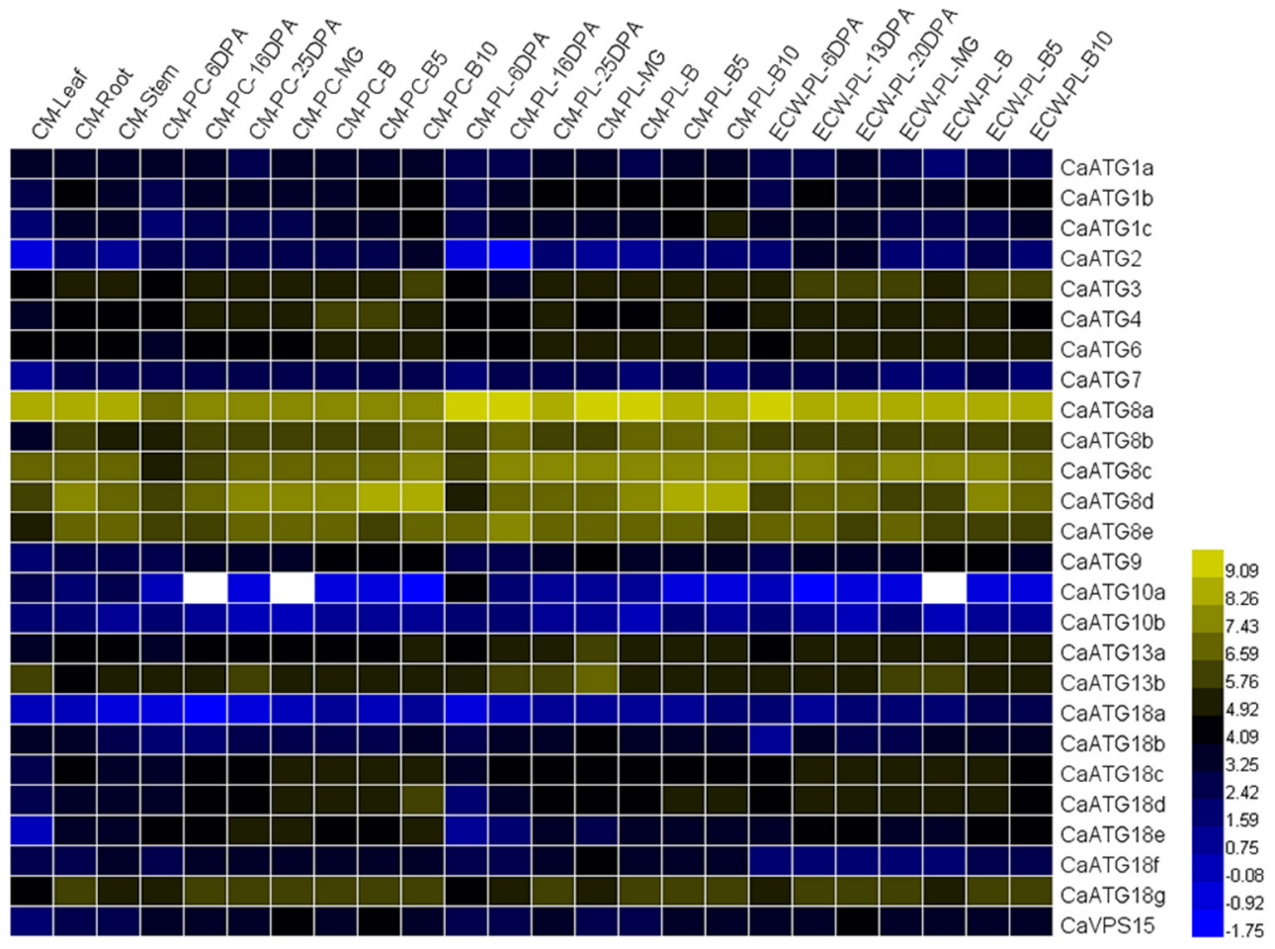
**RNA-seq analysis of *CaATG* genes tissue- and developmental stage-specific expression**. Raw data were retrieved from RNA-seq data of “CM334” (CM) and “ECW30R” (ECW). PC, pericarp; PL, placenta; DPA, days post anthesis; MG, mature green; B, breaker. The RNA-seq data were normalized using logarithm with the base of 2, and those of genes no transcription were indicated as white color.

To investigate the spatial and temporal expression patterns of *ATG* genes in pepper, we retrieved the data of 26 *CaATG* genes from the genomic RNA-seq databases of different tissues (root, stem and leaf) and fruit developmental stages (pericarp and placenta at various stages) of pepper cultivar “CM334” and “ECW30R.” As a whole, the expression of 26 *CaATG* genes showed tissue- and organ-specific pattern (Figure 3, Table S4), in which *CaATG2, 10a, 10b*, and *18a* showed a relatively low expression level, while *CaATG8a* and *CaATG8c* remained at higher expression levels in each tested tissue. Similar to the results in *Arabidopsis* (Sláviková et al., 2005), except *CaATG1a, 8a, 8c, 10a, 13b, 18b*, most of the *CaATG* genes were abundantly transcribed in root and stem, meaning the higher activity of autophagy in plant root and stem under normal growth condition. In addition, some *CaATG* genes, such as *CaATG3, 4, 6, 13a*, and *18g*, showed higher expression levels in reproductive organs, while *CaVPS15* only highly expressed in PC-MG, PC-B10 of “CM334” and PL-20DPA of “ECW30R.” Interestingly, with the increment in days after anthesis or breaker, a majority of the *CaATG* genes displayed up-regulated expression in pepper pericarp and placenta, suggesting the linkage of autophagy to the development and maturity of pepper fruit.

### Autophagy responses to abiotic stresses in pepper

**Figure 4:**
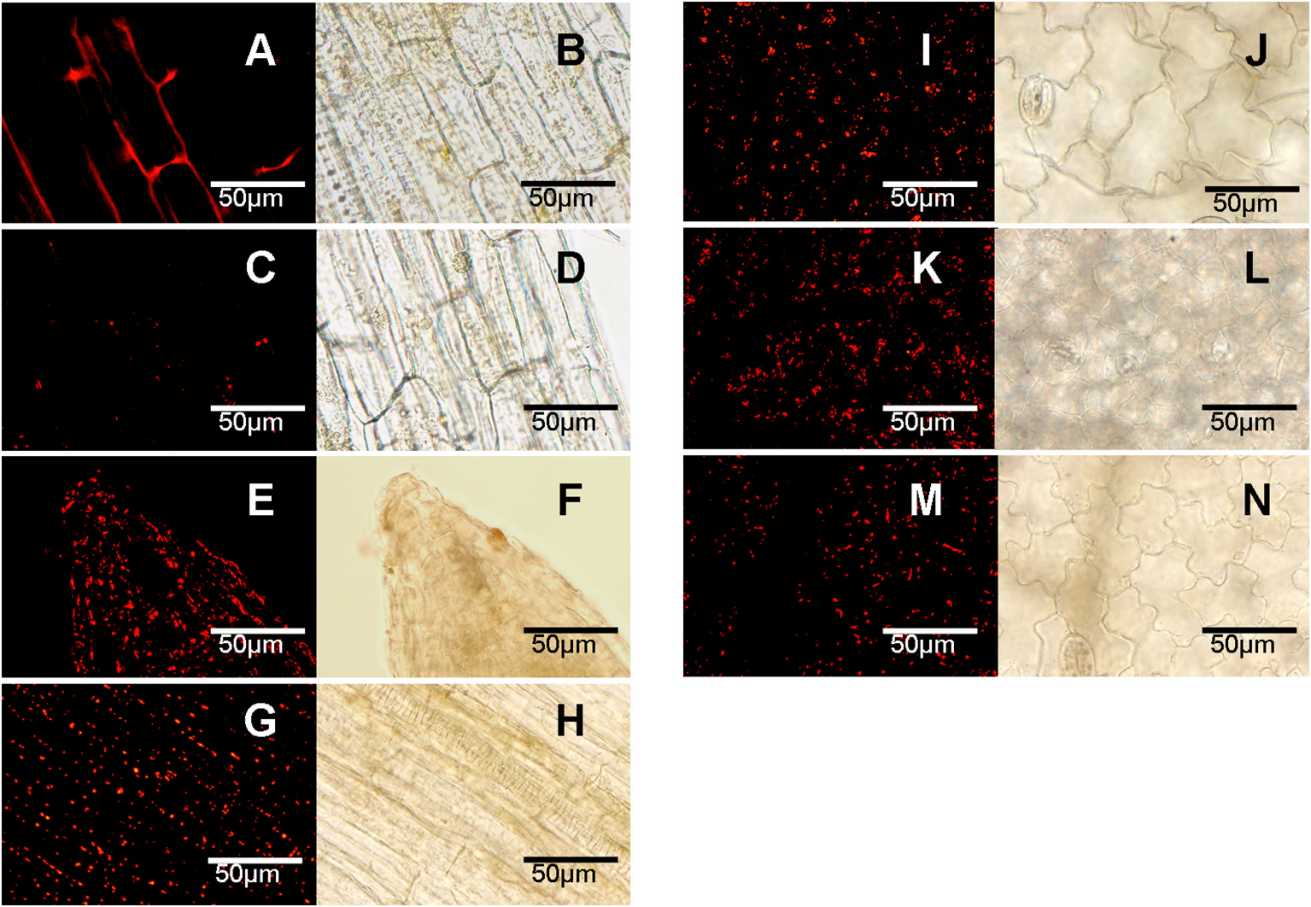
**Accumulation of autophagosomes under abiotic stresses (labeled with LysoTracker Red). (A–D)** Normal conditions; **(E,F)** salt stress of NaCl 3h; **(G,H)** drought stress of dried 3 h between folds of tissue pepper; **(I,J)** heat stress of 40°C 3 h; **(K,L)** cold stress of 4°C 3 h; **(M,N)** carbohydrate starvation stress of dark 2 days. **(A–H)** root; **(I–N)** leaf. **(A,B)** no E164d treatment; **(C–N)** infiltrated with E164d; **(A,C,E,G,I,K,M)**, stained punctates under fluorescence; **(B,D,F,H,J,L,N)**, under corresponding white light. The experiments were repeated three times with similar results. Scale bar = 50 μm.

The combination of LysoTracker Red as a probe and E-64d as an accumulator was used to detect the formation of autophagosome in pepper. When E-64d was absent, the punctate dots of autophagosome could not be accumulated (Figures 4A,B), while the punctates became visualized with the treatment of E64-d under both of normal (Figures 4C,D) and abiotic stresses conditions (Figures 4E-N). Compared to the less number under normal conditions (Figures 4C,D), punctate dots dramatically increased with abiotic stresses of salt (Figures 4E,F), drought (Figures 4G,H), heat (Figures 4I,J), cold (Figures 4K,L), and carbohydrate starvation (Figures 4M,N) in pepper leaves or roots.

**Figure 5:**
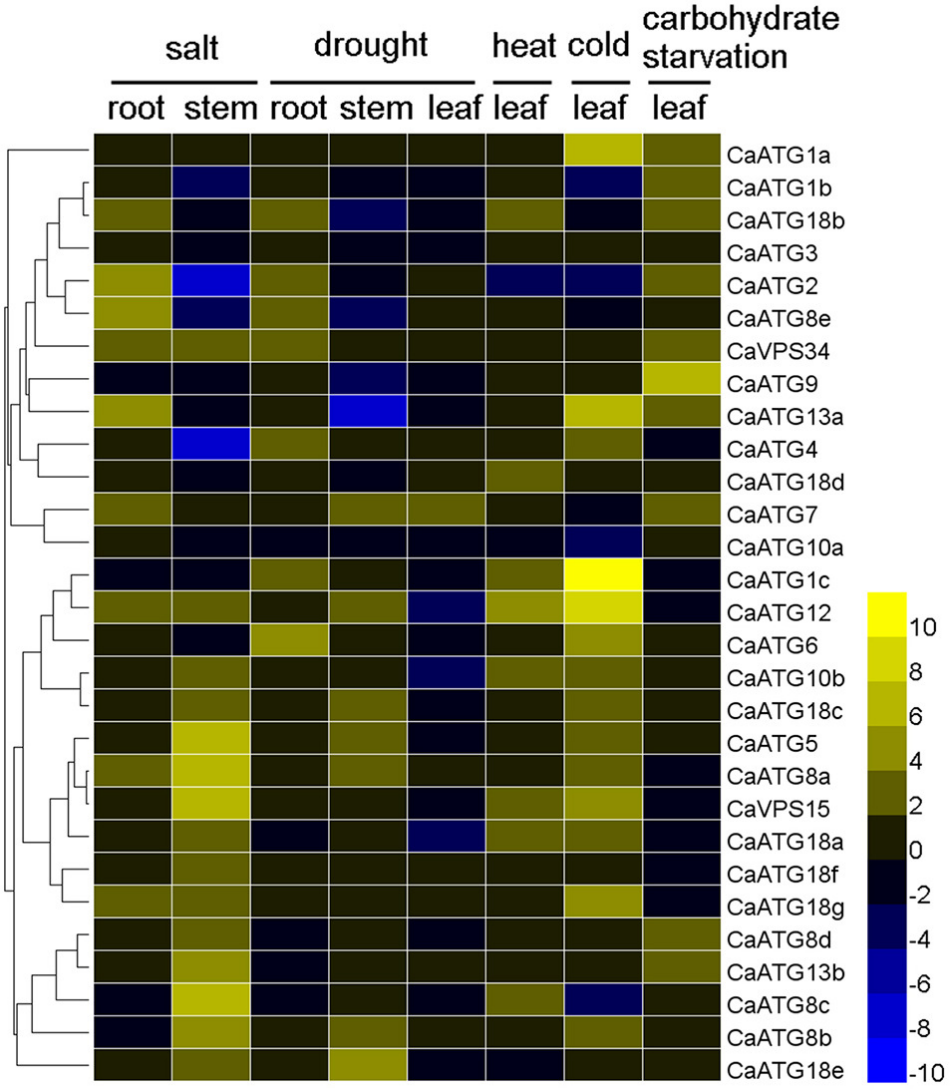
**Expression profiles of *CaATG* genes in response to abiotic stresses**. Pepper seedlings of R9 at 5–6 true leaves stage were used for salt (incubated in Hoagland's solution containing 200 mM NaCl for 3 h), drought (dried for 3 h between folds of tissue pepper), heat (kept at 40°C for 3 h), cold (kept at 4°C for 3 h), and carbohydrate starvation (kept in the dark for 2 days) treatments. The relative expression levels of pepper *CaATG* genes were calculated following the 2^−ΔΔCT^ method and the relative expression levels of the control seedlings were set to 1. In the heatmap, the values of 2^−ΔΔCT^ were used directly for the up-regulated genes, while the values of −(2^−ΔΔCT^)^−1^ were employed for the down-regulated genes. Expression data were obtained from three biological replicates.

The expression profiles of 29 *CaATG* genes responding to above abiotic stresses were further investigated thoroughly in sweet pepper line R9. The fold-change of *CaATG* genes expression levels under various stresses was shown in Figure 5 and Table S5. Out of 29 *CaATGs*, 16 *CaATG* genes (*CaATG1a, 1c, 5, 6, 7, 8a, 8b, 8d, 13b, 18c, 18d, 18e, 18f, 18g, VPS15*, and *VPS34*) were unaffected in some cases and up-regulated in others, and the expression of *CaATG3* did not show significant response to any stress treatment, while the rest of 12 genes showed stress-dependent changes patterns in their expression levels.

Under salt stress treatment, in pepper root, nine *CaATG* genes (*CaATG2, 7, 8a, 8e, 12, 13a, 18b, 18g*, and *VPS34*) were up-regulated, and no one showed down-regulation. In contrast, in stem, 15 genes (especially *CaATG5, 8a, 8c*, and *15*) were induced while four (especially *CaATG2* and *4*) were suppressed. For drought stress, the gene expression fold-change of *CaATGs* were lower in leaf than those in root and stem, and seven genes (*CaATG1c, 2, 4, 6, 8e, 18b*, and *VPS34*) in root, seven (*CaATG5, 7, 8a, 8b, 12, 18c*, and *18e*) in stem and one (*CaATG7*) in leaf were up-regulated, while four (*CaATG8e, 9, 13a*, and *18b*) in stem, three (*CaATG10b, 12*, and *18a*) in leaf, and no one in root were down-regulated, which was similar to that under salt stress. After heat stress, eight genes were significantly up-regulated but *CaATG2* was down-regulated. For cold stress, 14 *CaATG* genes particularly *CaATG1a, 1c, 12*, and *13a* were up-regulated, and four (*CaATG1b, 2, 8c, and 10a*) showed slightly down-regulation. As for carbohydrate starvation stress, 10 genes (*CaATG1a, 1b, 2, 7, 18d, 9, 13a, 13b, 18b*, and *CaVPS34*) especially *CaATG9* were up-regulated.

### Heat-induced autophagosome punctates accumulation and ATG genes expression in two pepper lines with different thermal tolerance

**Figure 6:**
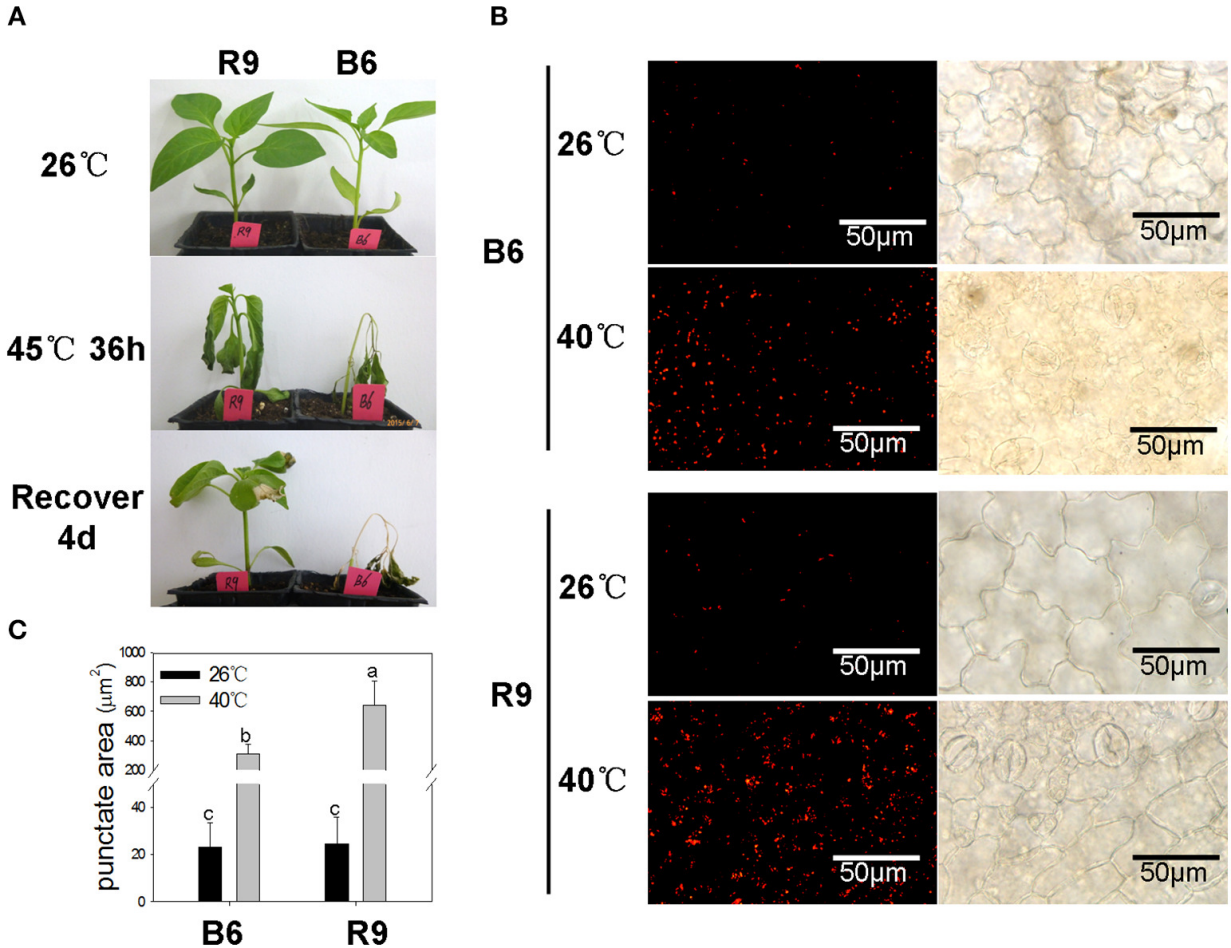
**Heat-induced autophagosome punctates accumulation in pepper**. R9, thermotolerant line; B6, thermosensitive line. **(A)** Phenotype observation of R9 and B6 seedlings under heat stress. When grown to 4–5 true leaves under normal temperature condition (26°C/20°C, day/night), the pepper seedlings were treated with 45°C for 36 h, and then transferred into normal conditions to recover for 4 days. **(B)** Accumulation of autophagosomes punctuates in pepper leaves under heat stress of 40°C 3 h. The experiments were repeated three times with similar results. Scale bar = 50 μm. **(C)** The punctate area of Lysotracker Red stained autolysosome-like structures in B6 and R9 leaves under normal conditions and heat stress. The different lower-case letters showed the difference of punctate areas between R9 and B6 under normal and heat stress conditions using the shortest significant ranges (*SSR*) method at the level α = 0.05.

As shown in Figure 6A, pepper line R9 showed higher ability in tolerance and restoration against heat stress than line B6. After 40°C heat treatment, the punctate dots were markedly induced in both lines (Figure 6B), and the area of fluorescent signals in R9 was significantly larger than that in B6 (Figure 6C), although the punctate accumulation did not show significant difference between R9 and B6 under normal temperature condition.

**Figure 7:**
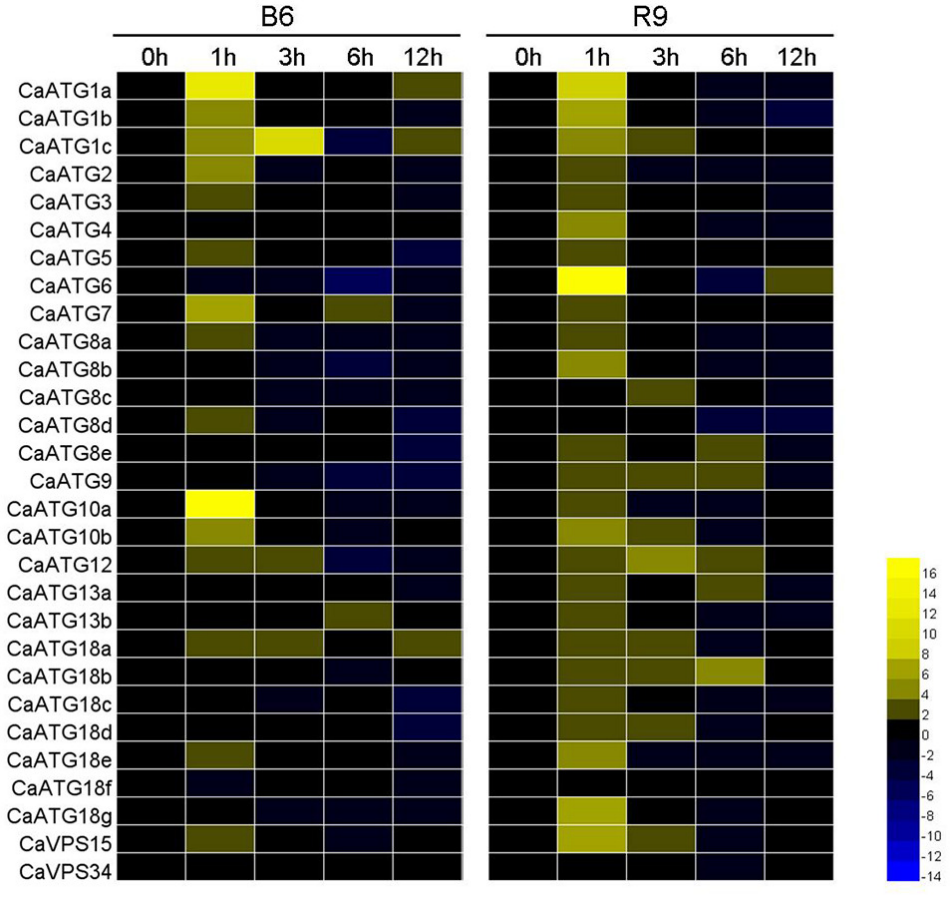
**Expression profiles of *CaATG* genes during heat stress in pepper leaf**. R9, thermotolerant line; B6, thermosensitive line. The relative expression levels of pepper *CaATG* genes were calculated following the 2^−Δ*ΔCT*^ method and the relative expression levels of the control seedlings at 0 h were set to 1. In the heatmap, the values of 2^−Δ*ΔCT*^ were used directly for the up-regulated genes, while the values of −(2^−ΔΔCT^)^−1^ were employed for the down-regulated genes. Expression data were derived from three biological replicates.

The time-course expression profiles of *CaATG* genes at 0, 1, 3, 6, and 12 h during heat stress were analyzed (Figure 7, Table S6). In leaves of pepper thermotolerant line R9, the expression of 26 out of 29 *CaATG* genes were up-regulated, while only *CaATG8d* and *VPS34* were weakly down-regulated, and no obvious change was observed in *CaATG18f*. Among the 26 up-regulated genes, 24 genes reached respective expression peaks at 1 h of heat stress, and *CaATG8c, 18b* at 3 and 6 h, respectively. The expression of almost all genes went down to their original levels at 12 h after heat stress treatment. For the thermosensitive line B6, 17 in 29 *ATG* genes were up-regulated especially *CaATG1a* and *CaATG10a*, and five genes were down-regulated. Among the 17 up-regulated genes, 16 genes showed the similar dynamic change patterns with those in R9, and only *CaATG8d* was up-regulated in B6 but down-regulated in R9. It is worth noting that compared with those in B6, 11 genes were specifically up-regulated in R9, among them five genes did not display obvious changes and six genes especially *CaATG6* were down-regulated in B6.

### Prediction of protein-protein interaction network

**Figure 8:**
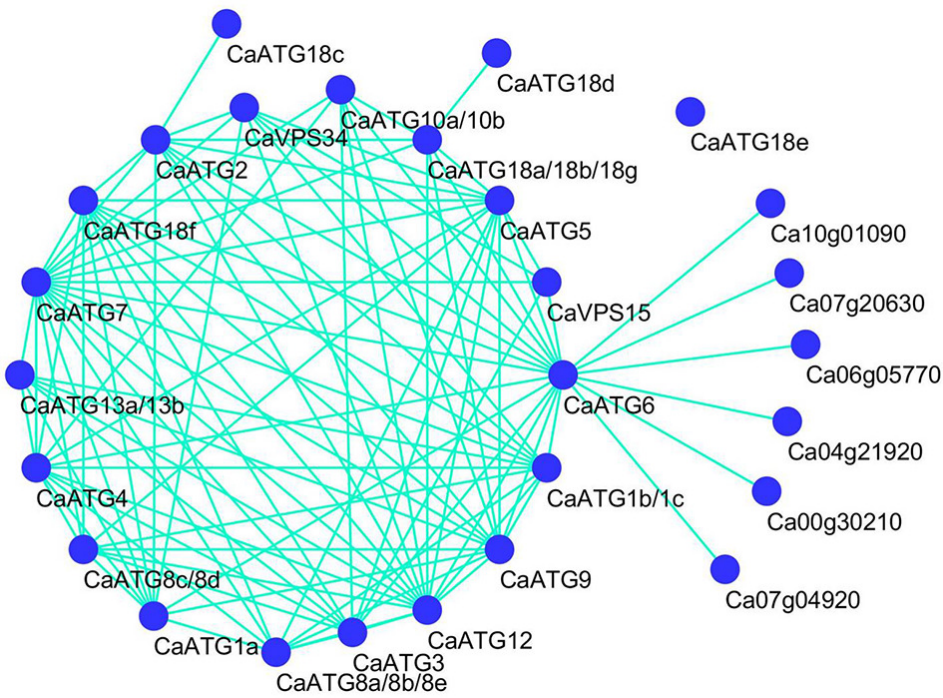
**Prediction protein-protein interation network among CaATG members and HSPs**. CaATG10a and 10b were both homologous with *Arabidopsis* AtATG10 (At3g07525.2), CaATG13a and 13b with AtATG13 (At3g49590.3), CaATG18a, 18b, and 18g with AtATG18a (At3g62770.1), CaATG1b and 1c with AtATG1b (At2g37840.1), CaATG8a, 8b, and 8e with AtATG8c (At1g62040.2), CaATG8c and 8d with AtATG8f (At4g16520.1), other CaATG proteins have single homolog each.

It is well known that the proteostasis controlled by the cooperation of autophagy and heat shock response is essential for heat tolerance of organisms (Dokladny et al., 2015), therefore the protein-protein interaction network of CaATGs and the interaction between CaATGs and CaHSPs were predicated based on the interolog from *Arabidopsis* (Figure 8). Among the CaATGs except CaATG18e (having no interaction partner), CaATG18c (only interacting with CaATG2) and CaATG18d (only interacting with CaATG18a/18b/18g), the other CaATGs possessed complex interaction networks, especially CaATG7 (having 17 interaction partners) and CaATG12 (15 interaction partners). Interestingly, CaATG6 could interact with almost all of other CaATG members except CaATG13 and CaATG8, furthermore, CaATG6 was predicted as a bridge to connect ATGs and HSPs, and all of the HSPs belonged to HSP90 family, including CA10g01090 (At5g56010.1, HSP81-3), CA07g20630 (At5g56000.1, Hsp81.4), CA06g05770 (At5g52640.1, HSP90.1), CA00g30210 (At2g04030.1, Hsp88.1), CA07g04920 (At3g07770.1, Hsp89.1), and CA04g21920 (At4g24190.1, Hsp90.7).

## Discussion

During recent years the autophagy, a ubiquitous process in eukaryotic cells for the degradation of damaged proteins and organelles, has been extensively investigated in the field of organisms fighting against disease or unfavorable environments (Klionsky, 2007). As a complex biological process, autophagy requires the pipelined collaboration of a set of ATG proteins with different functions (Chung, 2011).

In our study, a total of 15 core ATG members including 29 proteins with respective typical functional domains were identified based on the comprehensive genome sequencing data of pepper (Kim et al., 2014; Qin et al., 2014) (Table 1; Table S2), which is consistent with the 30 ATG proteins in *Arabidopsis* (Kwon and Park, 2008), 33 in rice (Xia et al., 2011), and 31 in maize (Li et al., 2015). In addition, same to the above three plant species, ATG14 and 27 are also missing in pepper, however, another absent ATG member ATG16 in pepper can be identified in the other three species, although the low sequence homology with its yeast counterpart, which suggests that the functional homologs of ATG16 maybe exist in pepper but characterize with higher diversity for identifying with regular BLAST research. Furthermore, it is noteworthy that unlike having two ATG4 proteins in *Arabidopsis* (Hanaoka et al., 2002), rice (Xia et al., 2011), maize (Chung et al., 2009), and wheat (Pei et al., 2014), only one ATG4 was identified in pepper, which is consistent with the result in tobacco (Zhou et al., 2015), suggesting a solanaceae crops-specificity in the number of ATG4.

Among the 15 ATG members in pepper, CaATG1, 8, 10, 13, and 18 are presented as gene families (Table 1), which are also observed in *Arabidopsis* (Kwon and Park, 2008), maize (Li et al., 2015), and rice (Xia et al., 2011). Although owning their respective conserved ATG domains (Table S2), same predicted subcellular localizations (except CaATG10a and 10b) (Table 1), in some degree, different proteins in the same CaATG members displayed differences in expression profiles during growth, development (Figure 3, Table S4) and responses to abiotic stresses (Figure 5, Table S5), suggesting multiple functions of homologous *ATG* genes (Sláviková et al., 2005), which may be related to the diversity in motifs and their distribution in ATGs proteins (Figures 2A,B, Table S3). For ATG1, the protein kinase domain (motif15-motif14-motif8) at N-terninal are conserved in the three CaATG1 proteins (Table S2), but the C-terninal regulatory domain (including motif19-motif20), functioning in forming a complex with ATG13 (Cheong et al., 2008), is missed in CaATG1a (Figures 2A,B), and this truncation is also observed in *Arabidopsis* (Suttangkakul et al., 2011) and maize (Li et al., 2015). Additionally, CaATG18 possesses a conservative N-terninal WD domain (motif13-motif4) in all 7 ATG18 proteins (Table S2), however, both CaATG18d and CaATG18e had a longer C-terninal extension (Figures 2A,B). These results suggest the possible functional divergence of different proteins in the same member, which need further research to verify.

Autophagy is widely involved in plants growth, development, and stress responses (Yoshimoto et al., 2004; Bassham et al., 2006), which is also supported by our results. According to the RNA-seq data published by Kim et al. (2014), the 26 *CaATG* genes expressed in a tissue- and developmental stage-specific patterns, in which some genes, like *CaATG8a* and *8c*, showed high expression levels in all the tested tissues and all the developmental processes of pepper fruit (Figure 3, Table S4). Under abiotic stresses of salt, drought, heat, cold, and starvation, the accumulation of autophagosome punctates increased markedly (Figure 4), showing the possibility of autophagy participation the pepper response to abiotic stresses. However, the expression levels of some *CaATG* genes were differently regulated by various stresses (Figure 5, Table S5). For instance, *CaATG1b* was induced by starvation but inhibited by salt and cold, and *CaATG2* was up-regulated by salt, drought and starvation but down-regulated by heat and cold, which were also observed in *CaATG8c, 10b, 12*, and *18b*. Furthermore, this differential regulation existed even in different tissues under the same stress. The expression of *CaATG8e* was enhanced in root but inhibited in stem by both of salt and drought, while in case of drought stress, the transcription of *CaATG12* and *18b* were induced in stem and root but inhibited in leaf and stem, respectively. These results indicate that autophagy may be regulated by distinct pathways during different abiotic stresses or in different tissues (Lv et al., 2014).

Compared to other abiotic stresses like drought, salt, starvation and cold, the roles of autophagy in heat stress are seldom reported in plant. The major negative consequence of high temperature on organism cell is protein misfolding and denaturation (Hemantaranjan et al., 2014), therefore, it can be hypothesized that autophagy plays an important role in plant heat responses. Zhou et al. (2013, 2014) found that heat stress can induce *ATG* genes expression and autophagosome punctates accumulation in both of *Arabidopsis* and tomato, and similar results were also observed in two pepper lines B6 and R9 in our study (Figures 6, 7). However, under heat stress condition, the number of up-regulated *CaATG* genes and the increment of punctate area in thermotolerant line R9 was obviously higher than those in thermosensitive line B6, which demonstrating an association of autophagy with heat tolerance in pepper.

In our previous and present research work, we have also found that under heat stress the expression of *CaHSP70-1* (Guo et al., 2014a), *CaHsf* family members (Guo et al., 2014b) were more abundantly transcribed in R9 than those in B6. Therefore, we speculate that pepper heat tolerance may be regulated by the cooperation between autophagy and heat shock response (HSR), the two aspects maintaining proteostasis, which is supported by the predicted interaction between autophagy (CaATG6) and HSR (CaHSP90 family) (Figure 8). Xu et al. (2011) reported that the functional interaction of HSP90 and ATG6 (Beclin 1) plays a role in controlling TLR (Toll-like receptor)-mediated autophagy in response to microbial infections, however, Dokladny et al. (2013) thought HSR can negatively regulate autophagy by HSP70 in mammals. Because HSP70 and HSP90 routinely work together in conducting their chaperone activities (Clerico et al., 2015), it is possible that ATG6, HSP70 and HSP90 jointly regulate autophagy in response to the changing environmental conditions. Additionally, due to the obvious up-regulation in R9 but down-regulation in B6 (Figure 7), CaATG6 is speculated playing a key role in the regulation of heat tolerance in pepper.

While the accumulation of dysfunctional proteins induced by adverse environmental condition cause damage to plants growth and development, efficient operation of protein degradation system including autophagy is necessary for survival. Combining the data presented in this study, we suggest an association between autophagy and pepper tolerance to heat and other abiotic stresses, which provides proof and reference for understanding plant tolerant mechanisms to environment stresses. However, future extensive researches are required to investigate the signal pathway to activate the autophagy process under unfavorable conditions.

## Author contributions

YZ, MG, and ML contributed to the experimental design. YZ, MG, HW, J. Lu, J. Liu, and CZ performed the research. YZ performed the bioinformatics analysis and drafted the manuscript. ML and ZG revised the paper and contributed reagents/materials/analysis tools. All authors read and approved the final manuscript.

## Funding

This work was supported by the National Natural Science Foundation of China (Grant No. 31572114), the Shaanxi Agriculture Science and Technology Projects (Grant No. 2014K01114101), the Basic Fund for Scientific Research of Northwest A&F University (Grant No. 2452015141), the Tang Zhongying Fund for Breeding of Northwest A&F University, and the Opening Fund of Key Laboratory for Crop Biotechnology of Xinjiang Uygur Autonomous Region (Grant No. XJYS030212014103).

### Conflict of interest statement

The authors declare that the research was conducted in the absence of any commercial or financial relationships that could be construed as a potential conflict of interest.

## Supplementary material

Table S1 **The *CaATGs* gene-specific primers for RT-qPCR**.

Table S2 **ATG domains of CaATG proteins in pepper**.

Table S3 **Motif sequences in CaATG proteins indentified by MEME tools**.

Table S4 **Expression data of *CaATGs* against RNA-seq data of each tissue**.

Table S5 **Expression data of *CaATG* genes in response to abiotic stresses**.

Table S6 **Expression data of *CaATG* genes during heat stress in peppe leaf**.

Figure S1 **Chromosomal localization of the 29 *CaATG* genes**.

## Abbreviations

HSPs: heat shock proteins
Hsf: heat shock transcription factor
ATG: AuTophaGy
GFP: green fluorescent protein
qRT-PCR: quantitative real-time PCR
HSR: heat shock response.
