# Supplementary material for: Autophagy, a Conserved Mechanism for Protein Degradation, Responds to Heat, and Other Abiotic Stresses in Capsicum annuum L
Source: Front Plant Sci. 2016 Feb 9;7:131. doi: 10.3389/fpls.2016.00131 (PMC4746239; doi:10.3389/fpls.2016.00131)
Supplement: Table S1 — The CaATGs gene-specific primers for RT-qPCR. [file Table1.PDF]

Table S1. The *CaATGs* gene-specific primers for RT-qPCR.

gene		Primer Sequence (5'→3')
CaATG1a	qCaATG1a-F	CGGTATGGAAAGCTGAGCAC
	qCaATG1a-R	CAAAGACATTGAAAAGGCGA
CaATG1ab	qCaATG1b-F	CACAGAAGCAGCCAGATGAGC
	qCaATG1b-R	TGAAGGACTACCAACAGGAACG
CaATG1c	qCaATG1c-F	CCTTGTATCTGGGTCCCTCAT
	qCaATG1c-R	GCACCGGCTCACCTAGTTTAG
CaATG2	qCaATG2-F	AAGGGAATGCTGGTTTTTATGT
	qCaATG2-R	AAGAGGTGGGATGGTAGTTTG
CaATG3	qCaATG3-F	TTTACCAGCCGACAAGCAAT
	qCaATG3-R	GCTCTCATCCTCAGCACCG
CaATG4	qCaATG4-F	AGATGCTTGTTGCTCAGGCT
	qCaATG4-R	CTTTTGCTGCGTGCTAATGTT
CaATG5	qCaATG5-F	CATAGTTGTGGACGACTTTTCA
	qCaATG5-R	TCTACCTGTTTTAGCTGGTGTTG
CaATG6	qCaATG6-F	AGAATCGTTTGTGGTTTTGC
	qCaATG6-R	ATCTGGGTCTGTGTTGTGGC
CaATG7	qCaATG7-F	TGTCTCTGCTGGTATGGTGAAC
	qCaATG7-R	GGTCTAGGGTACGGTTGGC
CaATG9	qCaATG9-F	TCTTGTCCTTGACCCGCAG
	qCaATG9-R	GAAGTCCGCAATGAAACGC
CaATG10a	qCaATG10a-F	TTGTATTGCCCATCTTTGGT
	qCaATG10a-R	CAAGATTTCCAGACTTATTGTGTG
CaATG10b	qCaATG10b-F	TTACGACTTTCATGTCATCTACAGT
	qCaATG10b-R	AAAGTCCTGTTCCAGGTCTTC
CaATG12	qCaATG12-F	TGTGAAGGGTGTGAGTGTGTG

	qCaATG12-R	AAGGCTAATGTTTGTCTCTGTTG
CaATG13a	qCaATG13a-F	ATCCTGGGCAGACTCGTTT
	qCaATG13a-R	CGGAATGACCTGATACCTGAAG
CaATG13b	qCaATG13b-F	ACATCCCATGAATCCCCTT
	qCaATG13b-R	TAGCACACCTGAGAACCGC
CaATG18a	qCaATG18a-F	ACTCTTTCACCTTTCTCCTCTCC
	qCaATG18a-R	GACTCTCATCATTTACCTGCTCG
CaATG18b	qCaATG18b-F	AGCCTCAGCCACAACAAGC
	qCaATG18b-R	TCTCAACAATTCCAATACCACCT
CaATG18c	qCaATG18c-F	GTTCAACTGTTTCACCTTCTCG
	qCaATG18c-R	GCTCCGTTTCATCACTGTCACT
CaATG18d	qCaATG18d-F	CAATCCACAGCACCTTACTCC
	qCaATG18d-R	GCAGCTCCTGATATCTTTACACG
CaATG18e	qCaATG18e-F	GAAAAGCCAGAACAACAGCAG
	qCaATG18e-R	GGAGCGAACATTAGACGACAC
CaATG18f	qCaATG18f-F	CTGTTCATGTTTTCTCTTTGGG
	qCaATG18f-R	GTTCAGGCGTTGCTGTCTC
CaATG18g	qCaATG18g-F	TCTACAACTGTGACCCGCTC
	qCaATG18g-R	CACCAACCAACGCCAAT
CaVPS15	qCaVPS15-F	GCGTCATTCAAACCCACC
	qCaVPS15-R	ATAACATCGCCTTCGTCCA
CaVPS34	qCaVPS34-F	GCGAAAATCAAGCAACCTC
	qCaVPS34-R	CGTCCAAGTCTAACCGAAAC
