# Supplementary material for: Autophagy, a Conserved Mechanism for Protein Degradation, Responds to Heat, and Other Abiotic Stresses in Capsicum annuum L
Source: Front Plant Sci. 2016 Feb 9;7:131. doi: 10.3389/fpls.2016.00131 (PMC4746239; doi:10.3389/fpls.2016.00131)
Supplement: Table S2 — ATG domains of CaATG proteins in pepper. [file Table2.PDF]

Table S2. ATG domains of CaATG proteins in pepper.

ATG members	pfam accession	family	description	ATG proteins	position
				CaATG1a	11-270
CaATG1	PF00069	pkinase	Protein kinase domain	CaATG1b	18-276
				CaATG1c	10-268
CaATG2	CaATG2	ATG2 CAD	Autophagy-related protein 2 CAD motif	CaATG2	294-346
CaATG3	PF03986	Autophagy_N	Autophagocytosis associated protein Atg3	CaATG3	7-166
CaATG4	PF03416	Peptidase_C54	Peptidase family C54	CaATG4	147-434
CaATG5	PF04106	APG5	Autophagy protein Apg5	CaATG5	84-354
CaATG6	PF04111	Apg6	Autophagy protein Apg6	CaATG6	185-694
CaATG7	PF16420	ATG7_N	Ubiquitin-like modifier-activating enzyme ATG7 N-terminus	CaATG7	10-327
				CaATG8a	14-117
				CaATG8b	14-117
CaATG8	PF02991	ATG8	Autophagy protein Atg8 ubiquitin like	CaATG8c	14-117
				CaATG8d	14-117
				CaATG8e	12-115
CaATG9	PF04109	APG9	Autophagy protein Apg9	CaATG9	199-563
CaATG10	PF03987	Autophagy_act_C	Autophagocytosis associated protein, active-site domain	CaATG10a	109-175

				CaATG10b	112-179
CaATG12	PF04110	APG12	Ubiquitin-like autophagy protein Apg12	CaATG12	123-195
CaATG13	PF10033	ATG13	Autophagy-related protein 13	CaATG13a	18-217
				CaATG13b	18-220
CaATG18	PF00400	WD40	WD domain, G-beta repeat	CaATG18a	66-322
				CaATG18b	65-318
				CaATG18c	29-294
				CaATG18d	370-468
				CaATG18e	368-468
				CaATG18f	164-251
				CaATG18g	82-343
CaVPS15	PF00069	Pkinase	Protein kinase domain	CaVPS15	27-294
CaVPS34	PF00454	Phosphatidylinositol 3- and 4-kinase	Phosphatidylinositol 3- and 4-kinase	CaVPS34	48-192
