# Supplementary material for: Autophagy, a Conserved Mechanism for Protein Degradation, Responds to Heat, and Other Abiotic Stresses in Capsicum annuum L
Source: Front Plant Sci. 2016 Feb 9;7:131. doi: 10.3389/fpls.2016.00131 (PMC4746239; doi:10.3389/fpls.2016.00131)
Supplement: Table S3 — Motif sequences in CaATG proteins indentified by MEME tools. [file Table3.PDF]

Table S3. Motif sequences in CaATG proteins indentified by MEME tools.

Motif	Width	Best possible match
1	50	RIREKYPDRIPVIVEKAEKSDIPDIDKKKYLVPADLTVGQFVYVVRKRIK
2	41	AIFIFVKNILPPTAAIMSAIYEENKDEDGFLYMTYSGENTF
3	50	PQYPRNKVMIWDDHQSRCIGELSFRSEVRAVRLRRDCIVVILEQKIYVYN
4	41	HLQEVRRGVDRAEIHSICFSPTAQWLAVSSDKGTVHVFALK
5	50	HQIETVANPKGLCEISQHAGSMVLVCPGLQKGQVRVEHYASNRTKFIMAH
6	36	YYVSFNQDYGCFAIGTDRGFRIYNCDPFCEMFRRYF
7	37	FIKGVLPKYFSSEWSVAQFRLPGCSQYIVTFGHQKNT
8	50	LAETLCGSPLYMAPEIMQLQKYDAKADLWSVGAILFQLVTGKTPFTGNNQ
9	29	GWDGSFYRCKFDPASGGEMTQLEYHNFLK
10	50	TIEDLEQDFPVYTAQRLAISKWTFITQEEHPYLNRPWYKLHPCGTSEWMK
11	50	WNKFDLGFPHWSWINYPKRPGFAASKVEGYLSLENMILPRSSEEDQHQCG
12	21	GGIGIVEMLFRCNILAFVGAG
13	21	GTLLATASTKGTLIRIFNTMD
14	29	KWFMQQLADGLKVLHNHNLIHRDLKPQNI
15	50	LKCEIVILQKINHPNIIRLHDMIEEPGKIYIVLEYCKGGDLSMYIQQRQG
16	41	KNLHPDCIDLCQKLLRCNPVERLTFEEFFNHPFLAQKQPDE
17	50	RWVVQCENRRTMVPQMGDSSYKKTYKKSIILLRSLYSMMRLLPAFKAFRK
18	15	MAKNSFKQEHPLEKR
19	50	IMELVNEKIEAGKHLEAFSIQLVILAIWKQALDICHMQAASAIEGSPNQE
20	49	AKHIEPGNAEVPDAMEMIFQSALEFGRKGAVDEYMGRTGDAVKFYSKAV

Motif numbers corresponded to the motifs in Fig. 2B.
