# Supplementary figures and images for: Autophagy, a Conserved Mechanism for Protein Degradation, Responds to Heat, and Other Abiotic Stresses in Capsicum annuum L
Source: Front Plant Sci. 2016 Feb 9;7:131. doi: 10.3389/fpls.2016.00131 (PMC4746239; doi:10.3389/fpls.2016.00131)

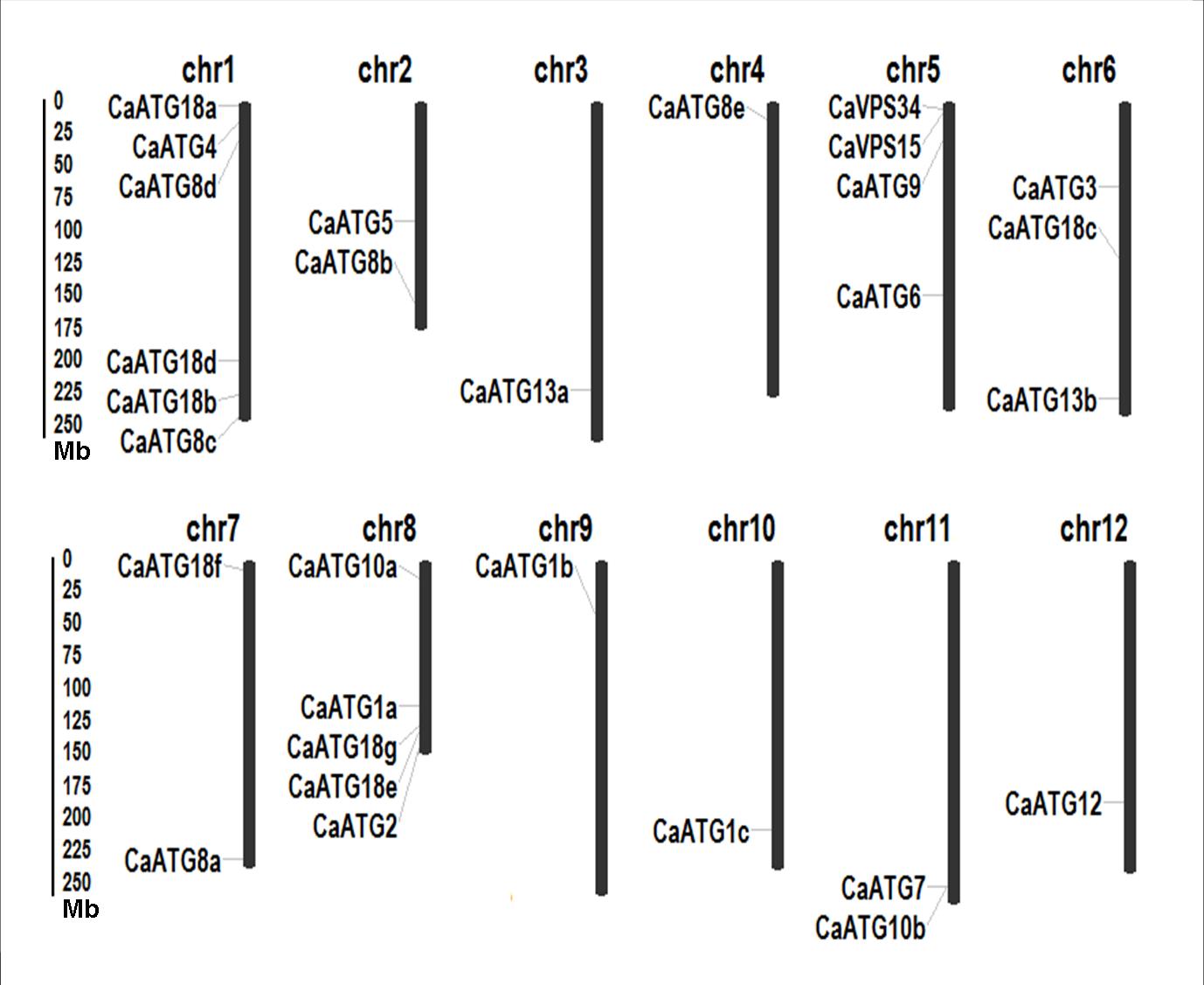

Supplement: Figure S1 — Chromosomal localization of the 29 CaATG genes. [file Image1.JPEG]
